# Timing and localization of human dystrophin isoform expression provide insights into the cognitive phenotype of Duchenne muscular dystrophy

**DOI:** 10.1038/s41598-017-12981-5

**Published:** 2017-10-03

**Authors:** Nathalie Doorenweerd, Ahmed Mahfouz, Maaike van Putten, Rajaram Kaliyaperumal, Peter A. C. t’ Hoen, Jos G. M. Hendriksen, Annemieke M. Aartsma-Rus, Jan J. G. M. Verschuuren, Erik H. Niks, Marcel J. T. Reinders, Hermien E. Kan, Boudewijn P. F. Lelieveldt

**Affiliations:** 1 C.J. Gorter Center for High Field MRI, Department of Radiology, Leiden University Medical Center, Leiden, The Netherlands; 2 Department of Neurology, Leiden University Medical Center, Leiden, The Netherlands; 3 Division of Image Processing, Department of Radiology, Leiden University Medical Center, Leiden, The Netherlands; 4 Delft Bioinformatics Lab, Delft University of Technology, Delft, The Netherlands; 5 Department of Human Genetics, Leiden University Medical Center, Leiden, The Netherlands; 6 Leiden Institute for Brain and Cognition, Leiden University, Leiden, The Netherlands; 7 Department of Neurological Learning Disabilities, Kempenhaeghe Epilepsy Center, Heeze, The Netherlands; 8 Department of Neurology, Maastricht University Medical Center, Maastricht, The Netherlands; 9 John Walton Muscular Dystrophy Research Centre, Newcastle University, Newcastle Upon Tyne, United Kingdom

## Abstract

Duchenne muscular dystrophy (DMD) is a muscular dystrophy with high incidence of learning and behavioural problems and is associated with neurodevelopmental disorders. To gain more insights into the role of dystrophin in this cognitive phenotype, we performed a comprehensive analysis of the expression patterns of dystrophin isoforms across human brain development, using unique transcriptomic data from Allen Human Brain and BrainSpan atlases. Dystrophin isoforms show large changes in expression through life with pronounced differences between the foetal and adult human brain. The Dp140 isoform was expressed in the cerebral cortex only in foetal life stages, while in the cerebellum it was also expressed postnatally. The Purkinje isoform Dp427p was virtually absent. The expression of dystrophin isoforms was significantly associated with genes implicated in neurodevelopmental disorders, like autism spectrum disorders or attention-deficit hyper-activity disorders, which are known to be associated to DMD. We also identified relevant functional associations of the different isoforms, like an association with axon guidance or neuron differentiation during early development. Our results point to the crucial role of several dystrophin isoforms in the development and function of the human brain.

## Introduction

Duchenne (DMD) and Becker (BMD) muscular dystrophies are X-linked genetic neuromuscular disorders characterized by severe and progressive muscle weakness. Mutations in the *DMD* gene result in absent/non-functional muscle dystrophin protein in DMD and shortened/partially functional protein in BMD.

In addition to skeletal muscle pathology, DMD is characterized by cognitive and behavioural problems with 30% of boys with DMD showing cognitive impairment (IQ < 70)^1^ and 40% having reading deficits similar to those observed in patients with phonological dyslexia^2–4^. Moreover, there is a higher incidence of attention-deficit/hyperactivity disorder (ADHD) (32%), anxiety disorder (27%), autism spectrum disorders (ASD) (15%), epilepsy (6.3%), and obsessive-compulsive disorder (OCD) (4.8%) in patients with DMD^5–7^. The progressive nature of the muscle pathology is a hallmark of the disease. Unfortunately, there are no longitudinal studies that investigate whether the cognitive pathology in DMD is progressive. Developmental delay was reported below the age of four and more cerebral atrophy was seen in men with DMD compared to boys, which may indicate progression with age^8,9^. However, normalization of IQ values in older patients has also been suggested^1^. In BMD patients, frequencies of learning difficulties or comorbidity with neurodevelopmental disorders have not been systematically reviewed. However, one report highlights a small group of relatively young patients, with spelling, arithmetic, reading difficulties, as well as behavioural problems and an earlier report had shown occurrence of epilepsy despite absent deviations from full scale IQ (FSIQ) distributions^10,11^. Caution is warranted, however, when projecting these results to the general BMD population.

**Figure 1:**
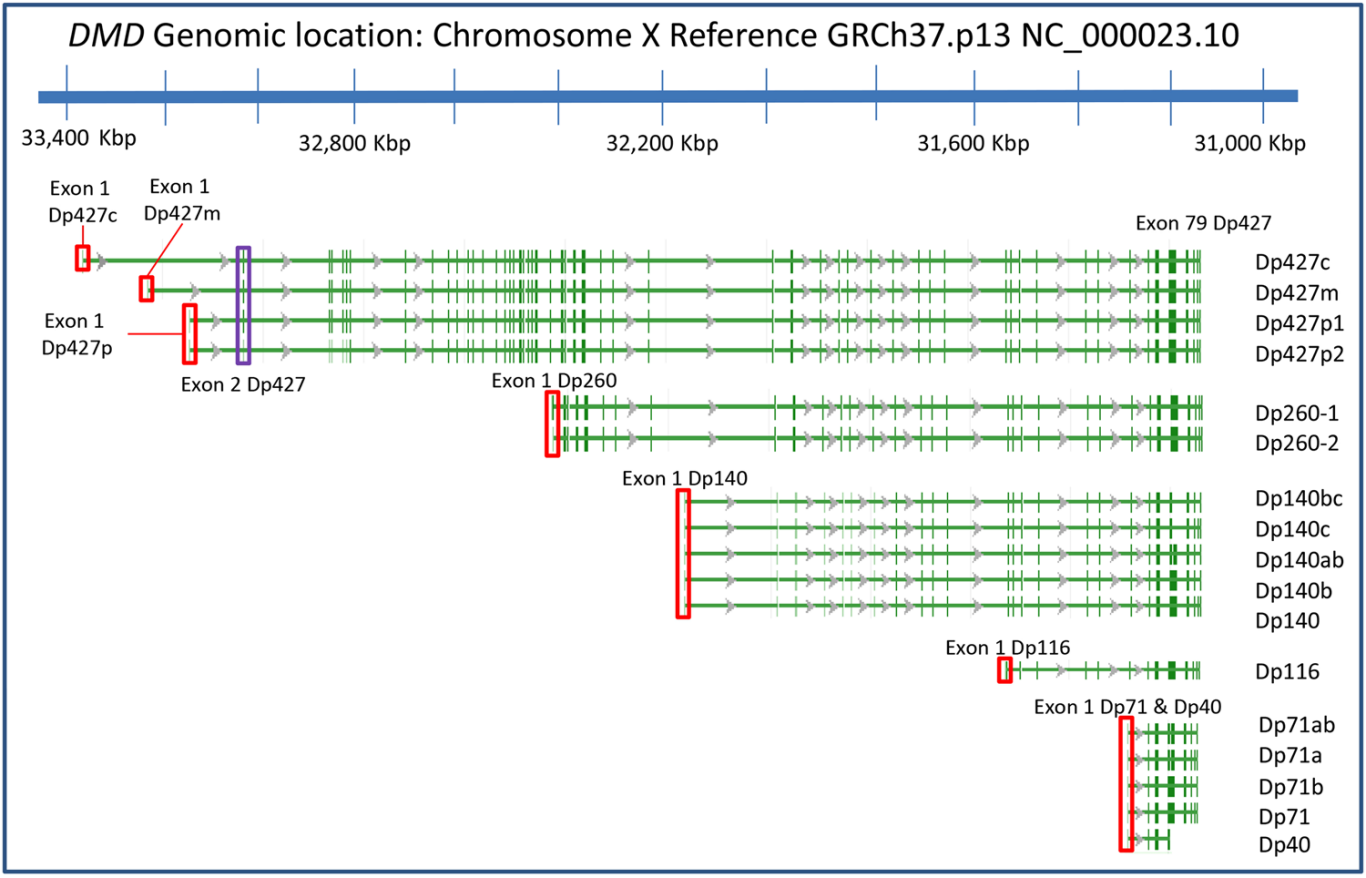
Human dystrophin isoforms. Dystrophin isoforms derived from the *DMD* gene located on the X chromosome (GRCh37.p13, RefSeq Release 74: NG_012232.1). The vertical green dashes indicate individual exons. The full-length dystrophins (Dp427) have 79 exons, with isoforms starting at unique first exons. For some isoforms, multiple splice variants have been identified (indicated on the right-hand side). The shorter isoforms (relative to the full-length isoforms Dp427) have unique first exons (i.e. not included in any other isoform), with the exception of Dp71 and Dp40 which share a first exon but use alternative polyadenylation sites. The red boxes indicate the position of the promoter region of each isoform. The second exon of Dp427c,m,p was used to represent the full-length dystrophin as a group (indicated by a purple box). This figure was generated using the NCBI’s Sequence Viewer.

The *DMD* gene contains at least seven independent, tissue-specific promoters and two polyA-addition sites, producing several isoforms that are named to reflect their length and splicing patterns (Fig. 1). The localization and function of the full-length muscle isoform Dp427m is well characterized both in humans and animal models. It is a crucial component of the dystrophin-glycoprotein complex (DGC), which bridges the inner cytoskeleton and the extracellular matrix providing structural stability to muscle fibers^12,13^. However, information on brain dystrophin is almost solely derived from animal models and cell culture studies, with only a few case studies in man^14,15^. It is believed that the cortical isoform Dp427c is predominantly expressed in neurons of the cortex and the CA regions of the hippocampus^16,17^. The Purkinje isoform Dp427p has two variants which are reported to be expressed in cerebellar Purkinje cells^18^. The shorter Dp260 and Dp116 isoforms are expressed primarily in the retina and the peripheral nerve, respectively^19,20^. There is very limited information on the sites of expression of the Dp140 isoform and its splice variants. A study of one 3.5 month old foetus and one 60 year old brain suggested that the Dp140 isoform is predominantly expressed during foetal life stages^21^. Finally, the Dp71 isoform is ubiquitously expressed, with higher levels in the central nervous system (CNS)^22,23^.

The risk of cognitive impairment in DMD has been associated with the location of mutations within the *DMD* gene resulting in the absence of specific dystrophin isoforms. FSIQ scores correlate with the number of isoforms missing. Patients missing all isoforms due to mutations in the distal part of the gene have the lowest scores, whereas patients missing only the full-length isoform have the highest scores^24^. Moreover, patients lacking Dp140 isoforms performed worse on all neuropsychological tests (general cognitive abilities, verbal memory, attention and executive functions) compared to those with preserved Dp140^25^. The relationship between the isoforms that are affected and the cognitive profile is further supported by the higher incidence of neurodevelopmental disorders in patients missing Dp140 compared to patients missing only Dp427^5,26^. This group distinction was already detectable below the age of four, as assessed with developmental quotients^8^. Finally, imaging has shown reduced grey matter volume and altered white matter microstructure compared to age-matched healthy controls, which was also more profound in patients missing Dp140^27^.

Despite this mounting evidence of the association between the absence of shorter dystrophin isoforms and higher incidence of learning and behavioural disabilities, the aetiology of the CNS pathology in DMD and BMD remains elusive. In this study, we provide detailed analysis of the spatial and temporal expression patterns of the dystrophin isoforms in the pathology-free adult and developing human brain. Using co-expression analysis, we characterize the functional role of the dystrophin isoforms as well as their relationships to other neurological disorders across brain development.

## Results

### Differential dystrophin isoform expression during brain development

**Figure 2:**
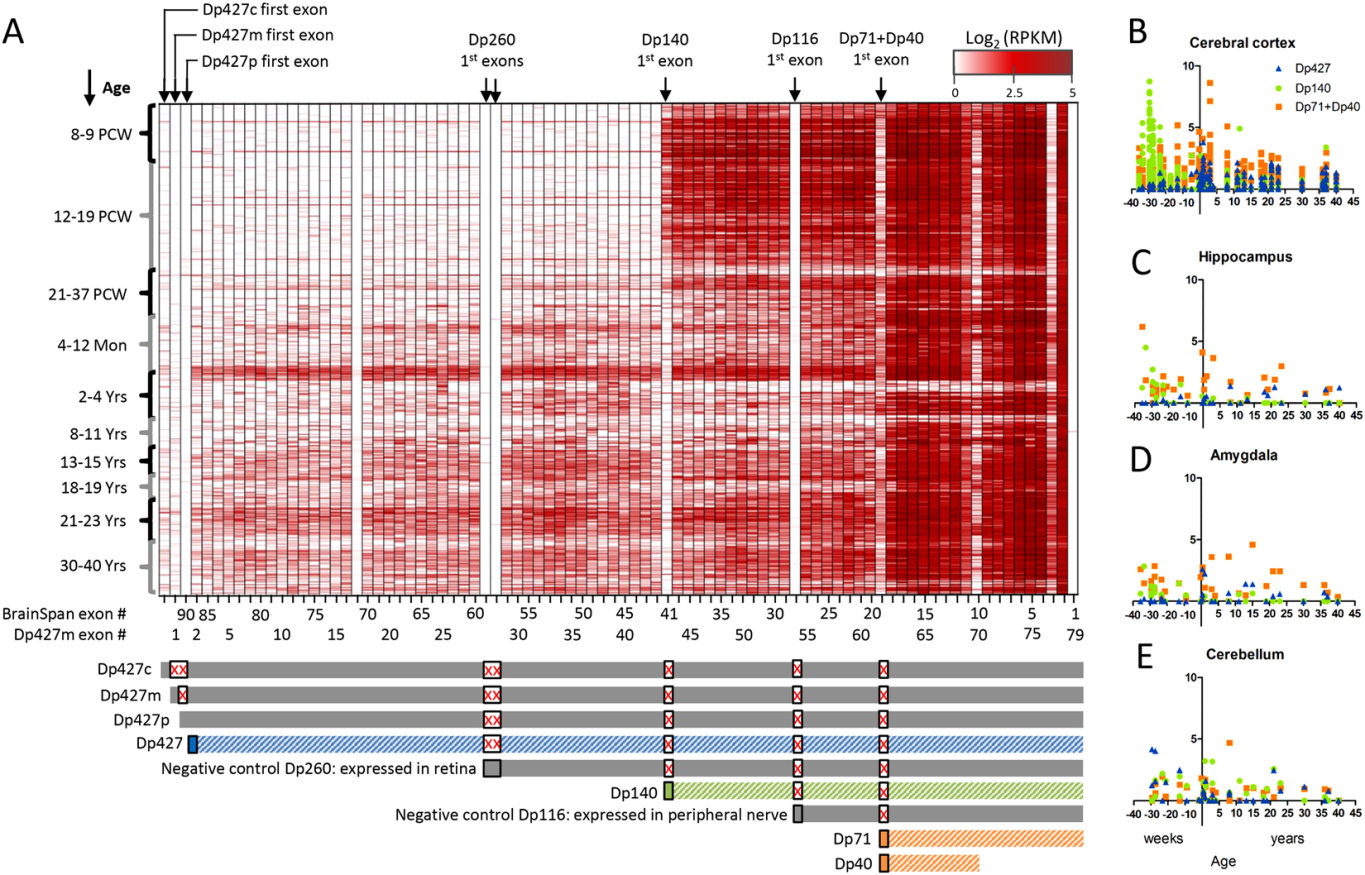
Dystrophin isoforms expression across brain development. (**A**) Dystrophin exons expression throughout brain development. The isoform unique first exons are indicated on top of the heatmap. The developmental stages are indicated on the left in post-conceptual weeks and months or years after birth. The BrainSpan atlas exon number is indicated below the heatmap together with the Dp427m exon numbering for reference. Bars below the heatmap indicate the different isoform groups. The grey bars corresponding to Dp427c,m,p are grouped together using exon 2 in further analysis (blue). The grey bars corresponding to Dp260 and Dp116 are expressed in the retina and peripheral nerves and are excluded from further analysis. The first exon of Dp140 (green) and Dp71 + Dp40 (orange) is used for further analysis. Boxes with a red ‘X’ indicate exons that are not part of the transcript. Expression values are presented as log_2_(RPKM). Brain region specific expression across development is shown for the cerebral cortex (**B**), hippocampus (**C**), amygdala (**D**) and cerebellum (**E**) of Dp427 (2^nd^ exon), Dp140 (1^st^ exon) and Dp71 + Dp40 (1^st^ exon). PCW: post-conception week, Mon: months, Yrs: years.

We used the BrainSpan atlas of the developing human brain transcriptome^28^ to assess the dystrophin isoform expression throughout development. The BrainSpan atlas provides RNA-sequencing expression profiling of 16 brain structures from 42 donor brains spanning early pre-natal development (8 weeks post-conception) to adulthood (40 years of age). In order to assess the expression of the different dystrophin isoforms, we used the expression of the unique first exons of Dp427p, Dp427c, Dp427m, Dp260, Dp140, Dp116 and the shared first exon of Dp71 and Dp40 (Fig. 2). We grouped the donors into 10 developmental stages (Supplementary Table 1). Figure 2 shows the expression of all exons within the *DMD* gene, across different brain regions and through development. The expression of Dp427c, and Dp427m is low during foetal development, shows a slight increase around the age of two, and is low throughout middle adulthood. This pattern is consistent across the different brain regions, though more prominent in the cerebral cortex when assessing Dp427 exon two, which is shared between Dp427p, Dp427c and Dp427m (Fig. 2B–D).

In contrast to previous reports^18,29^, the Purkinje isoform Dp427p was virtually absent in the brain throughout development, with expression levels even lower than muscle dystrophin Dp427m. To verify that Dp427p is indeed expressed in mouse, but not in human brain, we analysed the expression of Dp427p in cerebellum and cerebral cortex samples from control adult human brain (provided by the Netherlands Brain Bank) using quantitative polymerase chain reaction (qPCR). Indeed, we did not observe Dp427p expression in the human cerebral cortex and also not in the cerebellum, where the Purkinje neurons are located (Supplementary Figure 1). Yet in line with previous studies^30,31^, Dp427p was expressed in the mouse cerebellum and not in the mouse cerebral cortex (Supplementary Figure 1). This sharp contrast in Dp427p expression in the cerebellum suggests a different role for Dp427p in human than in mouse.

As expected, the samples representing retinal Dp260 and peripheral nerve Dp116 have virtually no expression in the brain (their unique first exons are not expressed). By contrast, Dp140 is clearly expressed in the foetal brain, with high expression in the early to mid-foetal stages, but very low expression from the late foetal stage onwards. Nevertheless, Dp140 is still expressed in the cerebellum and cerebral cortex at middle adulthood (n = 3), which has never been reported before (Fig. 2E). To verify that Dp140 is indeed expressed in the adult cerebral cortex and cerebellum, we analysed the expression of Dp140 as described above for Dp427p. Results confirm expression on Dp140 in the adult human cerebral cortex, as well as much higher expression in the adult human cerebellum (Supplementary Figure 1).

The Dp71+Dp40 expression is high during foetal stages and remains high after birth and later in life showing little regional specificity, in line with earlier reports indicating ubiquitous expression^14,32^. This is further supported by qPCR results showing comparable Dp71 expression levels in the cortex and cerebellum (Supplementary Figure 1).

### *DMD* expression in the adult human brain is high in the hippocampus and amygdala but low in the cerebellum relative to the brain average expression

To analyse the spatial distribution of *DMD* gene expression across the adult brain, we used the Allen Human Brain Atlas (AHBA), which has a much higher resolution than the BrainSpan atlas but lacks the temporal dimension. The AHBA provides microarray gene expression data from hundreds of samples extracted from six adult human brains, allowing detailed analysis of the regional expression of genes across the human brain. However, as oligo-dT primers were used for sample preparation, which capture the distal part of the gene, it is not possible to distinguish between different isoforms, nor is exon specific data available.

**Figure 3:**
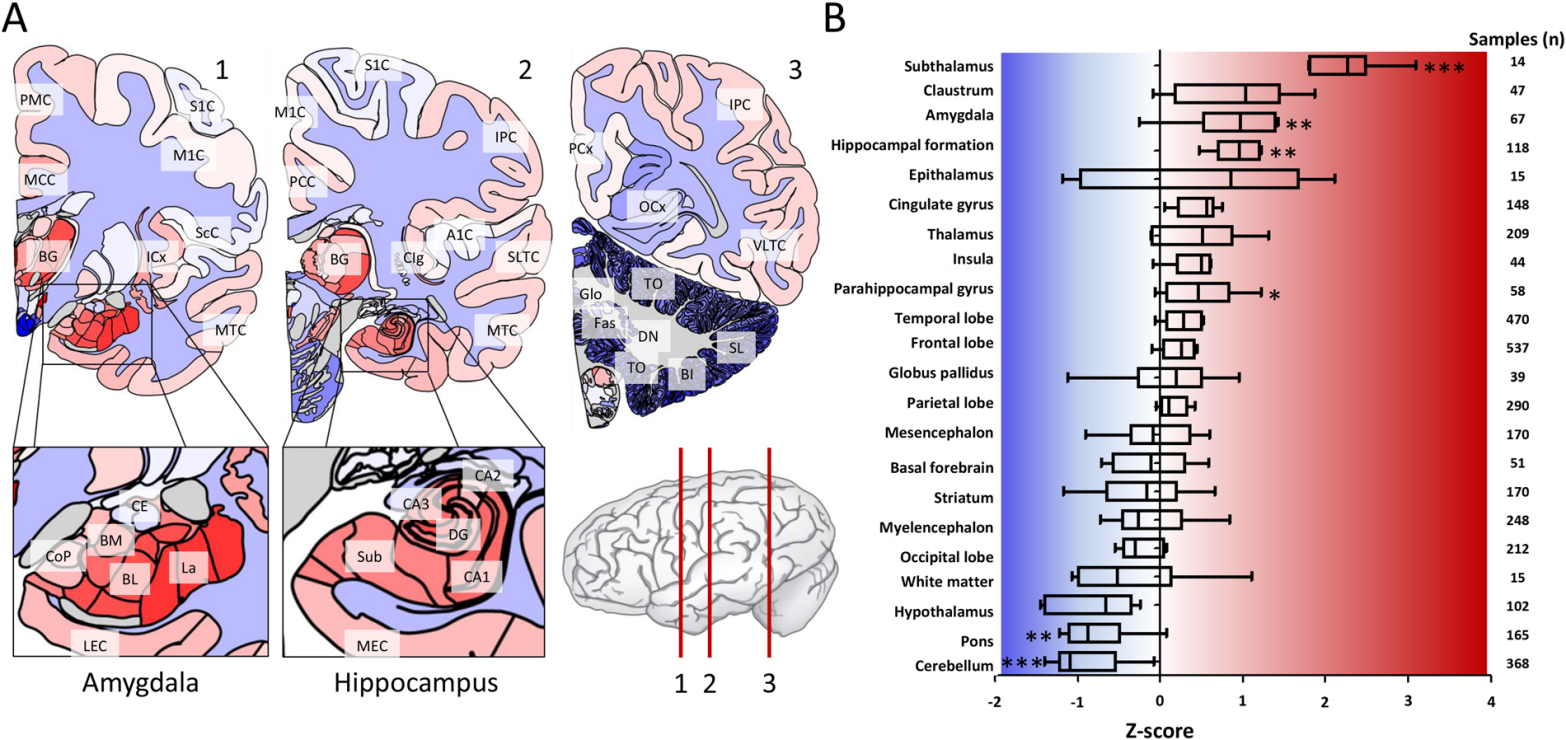
*DMD* gene expression across the adult human brain. *DMD* expression in the adult human brain at high spatial resolution averaged from six adult donors (five males and one female; mean age 42 years). Data is shown relative to the average expression across the whole brain (z-score normalization). (**A**) The spatial distribution is highlighted in three cross-sections of the brain showing the high sub-structural expression in the amygdala and hippocampus in contrast to the low expression throughout the cerebellum. (**B**) The brain was subdivided into 22 non-overlapping anatomical regions. For each region, the average expression in each of the donors was calculated separately (after z-score normalization) and all six average values are shown in a boxplot. The number of samples are indicated on the right. Significantly higher expression was found in the subthalamus, amygdala, parahippocampus and hippocampal formation. Significantly lower expression was found in the cerebellum and pons (Mann-Whitney U-test; *P < 0.05, **P < 0.01, ***P < 0.001). Full structure names for the indicated acronyms can be found in the Materials and Methods.

Relative to the average expression levels across the six donors, the highest expression levels of *DMD* were found in the hippocampus and amygdala (Fig. 3). Within the hippocampus, expression was highest in the CA4 region which is a small region in between CA3 and the dentate gyrus, and lowest in the CA2 region (Fig. 3A, Supplementary Table 2). The expression of *DMD* in the amygdala was highest in the basolateral complex (La, BL and BM), the input side of the amygdala that receives information from the prefrontal cortex, which is implicated in complex behaviour. Relatively low *DMD* expression was found on the output side with the central nucleus which connects with the brainstem and pons. Of the basolateral complex, highest *DMD* expression was found in the lateral nucleus which receives information from the neocortex, thalamus and hippocampus.

Animal studies have thus far consistently shown high dystrophin expression in the cerebellum^33,34^. Surprisingly, the lowest levels of *DMD* expression in the human brain were found in the cerebellum and the pons (Fig. 3B). Within the cerebellum, *DMD* expression was lowest in the globose (GL), fastigial (Fas) and dentate nuclei (DN) which receive inhibitory (GABAergic) input from Purkinje cells and excitatory (glutamatergic) inputs from mossy fibres and climbing fibre pathways. Second lowest expression was located in the regions associated with working memory, in the biventral lobule (Bl). The regions implicated in timing and coordination as well as attention through the prefrontal cortex, in the tonsilla (TO) and semilunal lobule (SL) were third lowest.

### Activity of transcription start sites in the *DMD* gene confirm expression observations

Gene expression is regulated by multiple factors that integrate at transcription start sites (TSSs) to control the transcription of target genes in a cell-specific manner^35^. To better characterize the activity of the dystrophin transcripts across different tissues and cell-types, we analysed TSS usage within the *DMD* gene across different brain regions. Genome-wide TSS usage has been detected across many human cell-types in the FANTOM5 consortium data set (using cap analysis of gene expression; CAGE)^36,37^ and by the Roadmap Epigenomic Consortium using chromatin markers specific to TSSs^38^.

**Figure 4:**
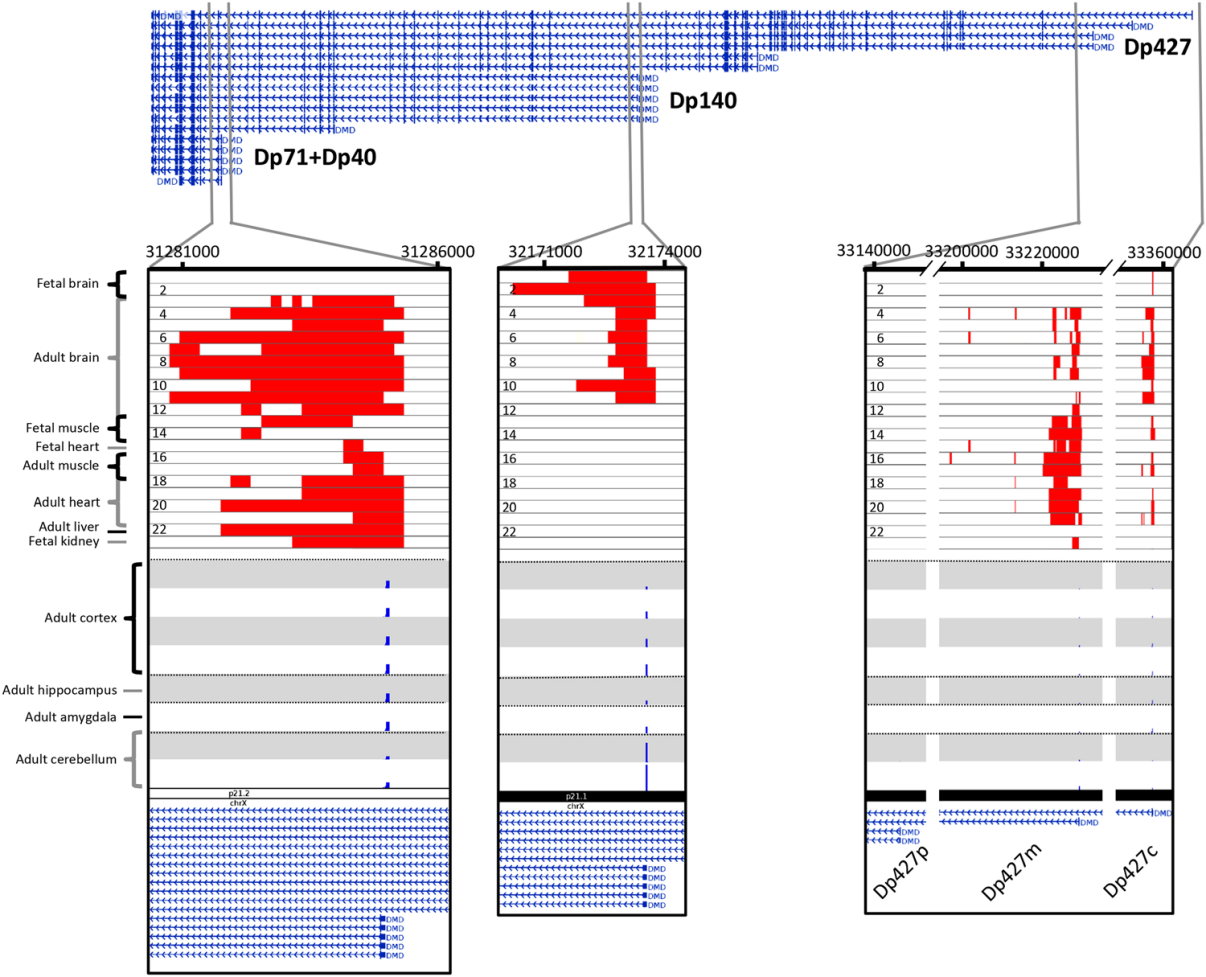
*DMD* transcription start sites. Genome browser view of the different TSSs within the *DMD* gene. Active TSS state based on histone markers within the *DMD* gene are shown for 23 samples including foetal and adult brain, muscle, heart, liver and kidney. See Materials and Methods for detailed sample information. Red bars indicate an active TSS state as defined by the Roadmap Epigenomic Consortium^38^. The bottom eight tracks show the TSS activity (blue bars) within the first exons of the different isoforms captured by CAGE sequencing from the FANTOM5 project^36,37^. All the active TSSs have been highlighted by zooming in on the first exons of the different isoform groups, from left to right: Dp71 + Dp40, Dp140, and Dp427. Note the absence of any TSS activity or epigenetic markers for Dp427p. Data is aligned to the human reference genome (GRCh37) and RefSeq transcripts are shown at the top. Data is plotted using the WashU Epigenome Browser^71^.

Using the FANTOM5 data, we mapped the usage of TSSs from tissue samples of the amygdala, hippocampus, cerebellum and cerebral cortex based on the TSS expression in the adult human brain (Fig. 4). In total, there were 25 TSSs within a window of 1 kb of the first exons of Dp427c, Dp427m, Dp427p, Dp260, Dp140, Dp116, and Dp70 + Dp41 (Supplementary Table 3). Consistent with our findings from the BrainSpan data and the qPCR experiment (Fig. 2 and Supplementary Figure 1), the TSSs of the Purkinje isoform were not expressed in any of the samples analysed. Similarly, we did not observe expression of the TSSs of Dp260 and Dp116. In addition, the expression of the TSSs of Dp427c was highest in the amygdala and hippocampus, in line with the observations from the AHBA analysis (Fig. 3). The short isoforms Dp71 + Dp40 were consistently expressed across the brain with lower expression in the cerebellum, in line with results from the BrainSpan analysis (Fig. 2A). The TSSs of Dp140 were expressed throughout the adult brain with higher expression in the cerebellum compared to the rest of the brain. The higher expression in the cerebellum is in line with the BrainSpan data (Fig. 2E). In contrast to the low expression levels observed after birth in the BrainSpan data, the expression levels of Dp140 TSSs were high in the cerebral cortex, hippocampus and amygdala of the adult brain. However, this matches our results from the qPCR analysis of the adult cerebral cortex and cerebellum (Supplementary Figure 1). Finally, the expression of the TSSs of the muscle isoform (Dp427m) was low across the brain except in the cerebellum.

To further investigate TSS usage within the *DMD* gene, we used the data from the Roadmap Epigenomic Consortium^38^ to identify TSSs based on their chromatin signatures. We analysed the chromatin signatures (i.e. chromatin states) across the *DMD* gene, focusing on active TSS, in adult and foetal brain samples as well as samples from the muscle, heart, liver, aorta, and kidney (see Materials and Methods for a full list of samples). In general, brain samples showed high TSS activity for the Dp140 and Dp71 + Dp40 isoform groups (Fig. 4), while the muscle and heart samples showed high TSS activity for the Dp427 isoforms group. The foetal brain samples showed active TSS at the first exon of the Dp140 isoform but no active TSS for the Dp71 + Dp40 isoform, supporting the expression patterns shown in Fig. 2. The Dp140 isoform contained active TSS markers in the Neurospheres Cortex Derived, Angular Gyrus, Germinal Matrix and Mid Frontal Lobe samples and no active TSS in the Substania Nigra, Anterior Caudate, Cingulate Gyrus, and Inferior Temporal Lobe samples.

### Dystrophin isoforms are significantly co-expressed with genes implicated in neurodevelopmental disorders

**Figure 5:**
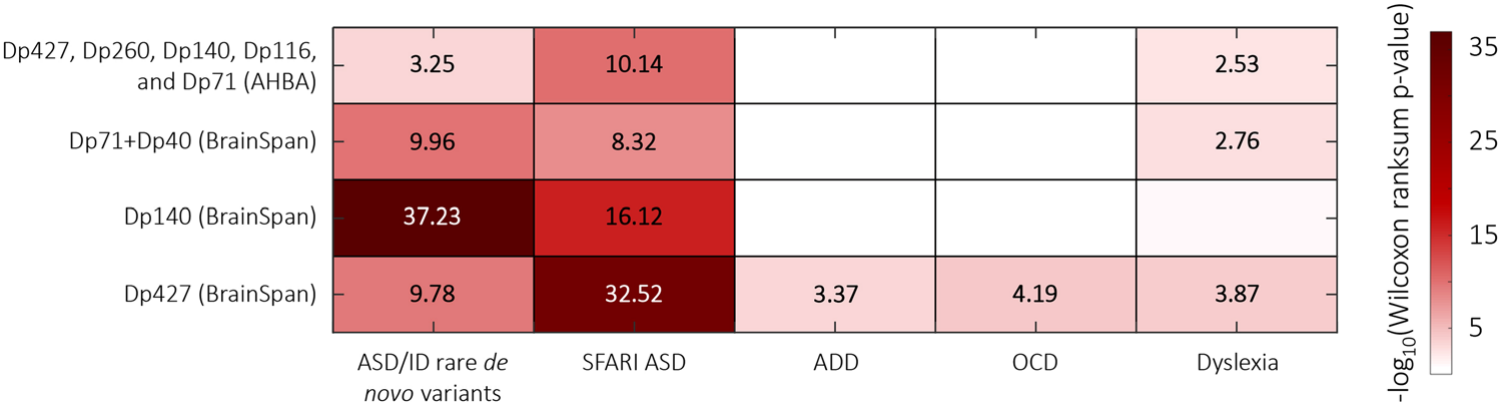
Genes co-expressed with dystrophin isoforms are enriched in disease-related genes. Genes co-expressed with *DMD* gene across the adult human brain as well as the dystrophin isoforms across brain development (rows) are analysed for enrichment in genes harbouring rare *de novo* variants in ASD and ID probands, a curated set of ASD risk genes (SFARI ASD), ADD-, OCD-, and dyslexia-related genes. Heatmap colours correspond to −log_10_(FDR-corrected *P*-value). All enrichment values for the lists enriched at *P* < 0.05 (one-sided Mann-Whitney U-test; FDR-corrected) are shown.

To get more insight into the functional role of dystrophin throughout human brain development and its association to other neurodevelopmental disorders, we analysed the spatial and temporal expression pattern relationships of the *DMD* gene and the different dystrophin isoforms. Co-expression analysis is a well-established approach to infer functional associations of genes using high-throughput expression data based on the ‘guilt by association’ principle^39^. First, we ranked all genes based on the correlation of their expression pattern to the *DMD* gene in the AHBA and to the three dystrophin isoform groups (Dp427, Dp140 and Dp71 + Dp40) in the BrainSpan atlas, resulting in four ranked gene lists (Supplementary Table 4). Next, we tested whether genes related to five disorders with high incidence in DMD patients (ASD, intellectual disability (ID), ADD, OCD, and dyslexia; Supplementary Table 5) are overrepresented among genes which are strongly co-expressed with *DMD* and the three isoforms (Fig. 5).

Genes associated with ASD and ID were significantly co-expressed with dystrophin expression patterns for both the full-length and shorter isoforms, especially Dp140 (FDR-corrected *P* = *5.66* × 10^−4^; one-sided Mann-Whitney U-Test; Fig. 5). In addition, ADD- and OCD-related genes were significantly co-expressed with Dp427 (FDR-corrected *P* = *4*.3 × 10^−4^; one-sided Mann-Whitney U-Test). The expression pattern of dyslexia-related genes followed *DMD* expression in the adult brain, as well as Dp427 and Dp71 + Dp40 expression in the developing brain (FDR-corrected *P* = *2.98* × 10^−3^; one-sided Mann-Whitney U-Test).

**Figure 6:**
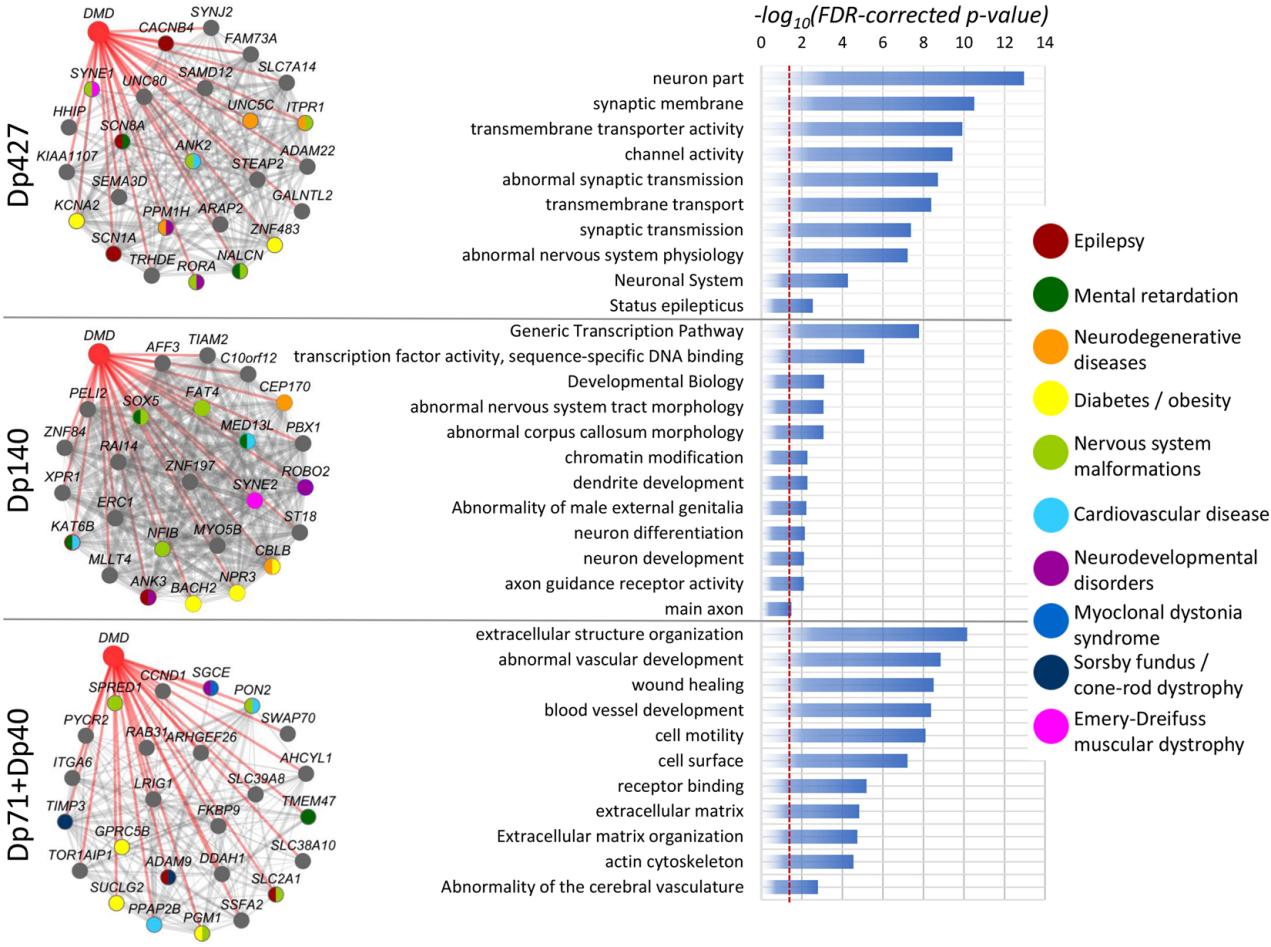
Dystrophin isoform co-expression networks and their associated GO-terms. Co-expression networks showing the top 25 positive correlating genes to the dystrophin isoforms Dp427, Dp140 and Dp71 + Dp40 across development. Edges between nodes represent correlation strength (the thicker the line the more correlated the genes). Correlations to dystrophin isoforms are shown in red (not weighted). Genes are color-coded according to their disease associations from DisGeNet. The bar plot shows the top terms enriched within the top 200 correlated genes. The vertical red line indicates the significance level (FDR-corrected *P*-value of <0.05).

To assess the strength of the connections between the top 25 of *DMD* associated genes, we mapped these based on their spatial and temporal co-expression patterns with Dp427, Dp140 and Dp71 + Dp40 in the developing human brain to a co-expression network. We combined this with the individual disease associations from DisGeNET^40^ to get an insight into the disease associations without prior selection based on previously reported co-occurrence in DMD (Fig. 6). The Dp140 network shows a stronger co-expression between genes compared to the networks of Dp427 and Dp71 + Dp40. The overlaid disease annotations show strong co-expression between dystrophin isoforms and other diseases such as epilepsy, mental retardation, obesity, nervous system malformation, neurodevelopmental disorders and cardiovascular problems. These spatial and temporal co-expression relationships point toward a functional association between *DMD* and genes related to these disorders.

To get an insight into the functional role of dystrophin throughout brain development, we assessed genes with strong co-expression to the three different dystrophin isoform groups for enrichment in gene ontology (GO) terms (Fig. 6, Supplementary Table 6). The molecular function associated to Dp427 was channel activity and transmembrane transporter activity. This matched with the biological process of transmembrane transport and synaptic transmission. The cellular component associated with Dp427 was the neuron part and more specifically the synaptic membrane. Finally, the phenotype associations were abnormal synaptic transmission and abnormal nervous system physiology.

Genes co-expressed with Dp140 were enriched in GO-terms related to early neurodevelopment via regulation of neuron differentiation and neuron projection morphogenesis as well as chromatin modification. The cellular component was the main axon and the top phenotype associations were abnormal nervous system tract morphology and abnormal corpus callosum morphology.

Finally, genes co-expressed with Dp71 + Dp40 were enriched in terms related to receptor binding as well as vascular development. The cellular component was the cell surface interacting with the extracellular matrix and the actin cytoskeleton and the top phenotype was abnormality of the cerebral vasculature.

## Discussion

We provide a comprehensive study of the expression of dystrophin isoforms in healthy human brain across anatomical regions and developmental stages. The detailed analysis of the expression patterns of the dystrophin isoforms and their spatial and temporal co-expression relationships provide a better understanding of the role of dystrophin in the development and function of the human brain.

The full-length isoforms Dp427c and even Dp427m show very low yet detectable expression throughout human development, confirming earlier reports^17,41^. However, the Purkinje isoform (Dp427p) showed almost no expression in the developing human brain data which is in contrast to an earlier study by Gorecki *et al*.^29^ which established the Purkinje specificity of Dp427p by showing its expression in the mouse cerebellum. A later report by Holder *et al*.^18^ showed expression of Dp427p in one adult cortical sample and no expression signal in a 20-week old foetal brain sample using PCR. To further confirm our findings from the BrainSpan atlas, we validated the absence of Dp427p expression from adult human and mouse cerebral cortex and cerebellar samples using qPCR and by interrogating TSS usage information from the FANTOM5 project. These results illustrate discrepancies in dystrophin isoforms expression between human and mouse brains and highlight the importance of comprehensive maps of expression in mouse and human brains for better translation between animal experiments to human conditions in which the *DMD* gene is implicated. It also means that results from studies specifically assessing the expression on Dp427p in mice may need to be reinterpreted^30,34^. Advances in single-cell RNA sequencing have made it possible to quantify gene expression levels in individual cells from different brain regions^42,43^. Mapping the expression of dystrophin isoforms in individual brain cells can greatly advance our understanding of the cell-type specific function of dystrophin in the brain and how the underlying cellular composition affects dystrophin function across brain regions.

The virtual lack of Dp260 and Dp116 expression confirms earlier hypotheses on the exclusive expression of these isoforms in the retina and peripheral nerves, respectively, despite a recent report indicating that Dp260 may be expressed in brain as well^44^. Based on the BrainSpan data, Dp140 is expressed mainly during the foetal stages of life across the brain, and at middle adulthood in few samples from the cerebellum and cortex. These results are in line with earlier results from foetal and adult human brain samples, where Western-blot analysis showed Dp140 in the foetal brain but not in the adult brain^21,32,33^. Interestingly, the AHBA data indicates that *DMD* expression is low in the cerebellum relative to the rest of the brain. Based on our validation we observed high expression of Dp140 in the adult human cerebellum and much lower expression in the cortex, in contrast to the earlier observation from AHBA. Further analysis of the expression of the Dp140 TSS from the FANTOM5 data indicates that indeed Dp140 is expressed/present in the adult human cerebellum.

The Dp71 and Dp40 isoforms showed consistent expression throughout brain development. Although we could not differentiate between Dp71 and Dp40 isoforms because they share a first exon, the high expression level of the last exon (belonging to Dp71 and not Dp40) suggests some specificity for the high signal to Dp71.

Caution is needed when extrapolating our results to protein quantity, as our results represent RNA expression levels which is still several steps removed from the final protein product^45^. Also, in the current study, we analysed the variable expression patterns of the different dystrophin isoforms across brain areas and development stages. Future work can determine the regulatory mechanisms responsible for these expression patterns. For instance, different isoforms might be regulated by different sets of transcription factors which can be discovered through a motif analysis and validated using chromatin immune precipitation followed by sequencing (CHIP-seq) experiments.

Cerebellar dysfunction has been suggested to underlie deficits in reading and verbal working memory as well as playing an role in ASD which are important components of the DMD cognitive deficit^46,47^. Our results rather emphasize the amygdala, involved in emotion regulation, and the hippocampus, involved in memory, based on their high expression of *DMD* and the supporting evidence of memory and emotion deficits in DMD from animal and neuropsychological studies in humans^5,26,48,49^. Moreover, we show a relatively even distribution of *DMD* expression throughout the cortex, involved in higher-order cognitive functioning. This is well in line with magnetic resonance imaging (MRI) studies demonstrating global structural alteration with reduced grey matter volume all through the cortex and altered white matter microstructure also throughout the brain in DMD patients compared to healthy age-matched controls^27,50^.

Co-expression analysis indicates a strong association between the dystrophin isoforms and genes implicated in ASD and ID, which might explain the high comorbidity of these disorders in DMD and possibly BMD patients. Functional enrichment analysis of strongly co-expressed genes advocates different functions for the three groups of isoforms throughout development. Dp140 shows a stronger co-expression within the network compared to the networks for Dp427 and Dp71 + Dp40. This may be related to the higher expression of Dp140 early in development. It could also be due to a physical link between the protein product of one of the neurodevelopmental disease genes to dystrophin. However, the protein domains within Dp140 largely overlap with those of the other dystrophin isoforms (with the exception of the N-terminal/actin binding domain which is specific to full length dystrophin). It is therefore unlikely that there are Dp140 specific protein interactions compared to Dp427 and Dp71, but it might be that there are cell-type specific interactions that are still undiscovered. The localization of Dp427 to the synaptic membrane in neurons with a function revolving around transmembrane transport and signal transmission further supports the proposed dystrophin-glycoprotein complex-like structure positioned in post-synaptic densities of GABA-ergic neurons in the brain^14^. Dp140, however, clearly shows a different role tailored towards axon guidance and transcription factor activity as well as neuron differentiation which are early neurodevelopmental processes. Absence of Dp140 may therefore lead to abnormal nervous system tract morphology as proposed by the GO-terms which might explain the altered white matter microstructure detected by MRI^27^. The presence of Dp140 in later life is unexpected as the main functional terms relate to early neurodevelopment. However, transcription factor activity or chromatin modifications, which were also strongly associated, may suggest a role in generic transcription pathways. It is worth noting that mutations in genes involved in chromatin remodelling are strongly associated to ASD^51^.

The association of Dp71 + Dp40 signal transduction plus transmembrane receptor binding in relation to growth factors implies that the structural alterations thus far observed in the brains of patients missing Dp427 and Dp140^27^ may be further aggravated in patients missing Dp71 and Dp40. Future studies on cerebral structural integrity in the absence of Dp71 and Dp40 can test this hypothesis. Interestingly, Dp71 + Dp40 were also associated with the cerebral vasculature and blood vessel development. Studies have shown altered cerebral blood flow in DMD, but the mouse models used in these studies did not have mutations that were predicted to affect Dp71 of Dp40^52^.

Clinical trials for DMD include strategies that aim to restore functional dystrophin protein targeting transcript pre-mRNA splicing or translations^53–57^. In order to restore function of dystrophin in the brain, compounds need to cross the blood-brain-barrier (BBB), which is not the case for all. However, the implications of restoring dystrophin in the brain if they do cross the BBB are not clear, which argues that preclinical assessments and clinical trials need to monitor the central nervous system.

Our results indicate that dystrophin isoforms play an important role in the development and function of the human brain. These findings advocate for the necessity to profile the expression of the dystrophin isoforms in post-mortem brain samples from people with dystrophinopathies in order to elucidate the transcriptional mechanisms underlying the behavioural and learning problems in DMD. This can greatly facilitate risk assessments of comorbid disorders and guide screening for early detection and targeted treatment. It also argues for the need of clinical trials to target and monitor the central nervous system.

## Materials and Methods

### BrainSpan developing human brain transcriptome

RNA-sequencing-derived exon-level expression data of the different isoforms of DMD was downloaded from the BrainSpan atlas of the developing human brain transcriptome^28^. RNA sequencing (RNA-seq) data was generated from 524 tissue samples collected from 42 post-mortem brains collected from neurologically unremarkable individuals spanning early pre-natal development (8 post-conception weeks, PCW) to late adulthood (40 years of age). Samples were extracted using macro dissection from 8–16 regions per brain. Details of tissue acquisition and data processing can be found on the website (http://www.brainspan.org). Gene and exon annotation of the RNA-seq data was derived from Gencode version 10 (GRCh37 – Ensembl 65). The expression level of the exons was measured in RPKM (reads per kilobase of exon model per million mapped reads).

The *DMD* gene (Chromosome X: 31,115,794–33,357,558) data included 94 exons. There are however 79 exons in the well-known muscle dystrophin Dp427m (NM_004006.2). We therefore mapped the exon locations to the Dp427m exon annotation (Fig. 2). In this process, we found the values specific for the isoform first exons (which are not exons in Dp427m) and we found genomic coordinates that were mapping to a lymphocyte isoform (Dp427l). This isoform is no longer included in the latest release of the human genome (Gencode version 21; GRCh38 – Ensemble 78), due to lack of evidence^58^. As such, we excluded these exon coordinates from further analysis.

### Adult human brain expression data

Spatial gene expression data from six adult human brains was obtained from the Allen Human Brain Atlas database (AHBA)^59^. Samples were collected from post-mortem brains from five males and one female between 24 and 57 years of age (mean age 42), with no known psychopathologies, by either manual macrodissection (cortical and some subcortical structures) or by laser-based microdissection (subcortical and brainstem areas). For each brain, RNA was extracted from 363 to 946 different samples per brain (3702 samples in total) and measured on custom Agilent microarrays containing the 4 × 44K Agilent Whole Human Genome probes as well as an additional 16,000 custom probes. For genes with two probes, the one with the highest variance was selected. For genes with at least three probes, the connectivity of each probe was calculated (sum of the Pearson correlations to all other probes, measured per brain and then averaged) and the one with the highest connectivity was selected. Expression data of the 19,991 genes was z-score normalized per brain. The expression of the *DMD* gene was measured using six probes, of which A_24_P185854 (NM_004023.1) has the highest connectivity and hence was used for further analysis. This probe is located at the distal part of the gene and captures the Dp71, Dp116, Dp140, Dp260, and Dp427 isoforms (all except Dp40). For visualization, z-score values of the *DMD* gene expression were mapped to anatomical atlas images acquired from the Allen Human Brain Atlas^59^. The following acronyms are indicated in Fig. 3A; BG: Basal ganglia, BL: Basolateral nucleus, BM: Basomedial nucleus, BL: Biventral lobule, CA1: CA1 region of the hippocampus, CA2: CA2 region of the hippocampus, CA3: CA3 region of the hippocampus, Cig: Caudal granular insular cortex, CE: Central nuclear group, DG: Dentate gyrus, DN: Dentate nucleus, Fas: Fastigial nucleus, Glo: Globose, ICx: Insular neocortex, LEC: Lateral entorhinal cortex, La: Lateral nucleus, MEC: Medial lateral entorhinal cortex, MCC: Midcingulate gyrus, MTC: Midlateral temporal cortex, OCx: Occipital neocortex, PCx: Parietal neocortex, PCC: Posterior cingulate cortex, CoP: Posterior cortical nucleus, IPC: Posteroventral parietal cortex, IPC: Posteroventral parietal cortex, PMC: Premotor cortex, A1C: Primary auditory cortex, M1C: Primary motor cortex, S1C: Primary somatosensory cortex, SL: semilunal lobule, SsC: Subcentral cortex, Sub: Subicular cortex, SLTC: Superolateral temporalcortex, TO: Tonsilla, VLTC: Ventrolateral temporal neocortex.

### Co-expression Analysis

To characterize the functional association of the *DMD* gene in the adult human brain, we calculated the spatial correlation (Pearson’s) between each gene in the AHBA (19,991 genes) and the *DMD* gene using all samples concatenated from the six donors (3,702 samples). Genes were ranked based on the correlations in a descending order. To assess the functional association of the different dystrophin isoforms across development, we calculated the spatial-temporal correlation (Pearson’s) between each exon in the BrainSpan dataset (241,690 exons) and the exons that are specific to each of the three dystrophin isoforms groups: exon 2 for full-length Dp427, isoform specific exons located in intron 44 for Dp140 and intron 62 for Dp71 + Dp40 with respect to Dp427m nomenclature. These isoforms were selected because virtually all DMD patients have mutations affecting Dp427c, Dp427m and Dp427p^60^. A proportion of patients additionally cannot produce Dp140. A small number of patients cannot produce any isoforms, including the shortest Dp71 and Dp40. For each isoform group, we ranked all exons in a descending order based on correlation. To get a ranked gene list, each gene was assigned the rank of its most correlated exon. For each gene set, functional enrichment analysis was performed on the top 1% (most positively correlated). Functional enrichment analysis was performed using ToppGene^61^. We returned all terms enriched at a false discovery rate (FDR)-corrected q-value of 0.05 from the categories: GO Molecular Function, GO Biological Process, GO Cellular Component, Human Phenotype, Mouse Phenotype, and Pathway.

### Disease gene sets overrepresentation

Enrichment analysis of disease-related gene sets was performed using a two-sided Wilcoxon rank sum test (Mann-Whitney U-test). For each list of all genes ranked based on their co-expression with dystrophin expression we used the rank sum test to assess the significance of the ranks of each disease gene set. To control the FDR, we corrected for multiple testing using the Benjamini-Hochberg method^62^. In case of the adult human brain we tested the set of 19,991 genes, ranked based on their correlation to the DMD gene across all samples (Pearson’s correlation). Similarly, for the BrainSpan developing human brain transcriptome we tested three sets of 21,164 genes ranked based on their co-expression with the exons corresponding to the three dystrophin isoforms: Dp71 + Dp40, Dp140 and Dp427.

We tested for the enrichment of five disease-related gene sets. The ASD-ID list contained 827 genes harbouring *de novo* mutations from four ASD^63–66^ and two ID^67,68^ exome sequencing studies. The ASD-ID was retrieved from^69^. The SFARI ASD list contained 706 genes associated to ASD using manual curation of published scientific literature from the Simons Foundation Autism Research Initiative (SFARI) AutDB database^70^. The list includes candidate genes implicated by common variant association, candidate gene studies, genes within ASD-associated copy number variants (CNV), and genes implicated in syndromic forms of ASD. Lists of genes related to ADD, OCD and dyslexia were retrieved from DisGeNet v3.0, a database that integrates human gene-disease associations from various expert curated sources and text-mining of literature^40^.

### Fantom5 data

We used the FANTOM5 samples ontology and the linked data version of FANTOM5 data, which was exposed as nanopublications^36,37^. We queried the FANTOM5 data to get all transcription start sites which are overlapping with the first exons of Dp427c, Dp427m, Dp427p, Dp260, Dp140, Dp116, and Dp70 + Dp41. We selected only samples belonging to the cerebral cortex, hippocampus, amygdala and cerebellum brain regions. Further, we removed samples pooled from multiple donors since they spanned a wide age range, which could dilute the expression of a transcription start site (TSS) varied through development. Our analysis resulted in 25 TSSs across eight samples (Supplementary Table 3).

### Epigenetic data

Data from the Roadmap Epigenomics Consortium^38^ was visualized using the WashU EpiGenome Browser v40.0.0^71^. We visualized only the track corresponding to active TSS chromatin state. Details of the 23 selected samples are in Supplementary Table 7.

### Validation using *ex vivo* qPCR

Frozen tissue samples from a 51-year-old male non-demented control brain of the anterior orbital gyrus and cerebellum were obtained (post-mortem delay: 07:45 hr; pH 7.05, stored in cryovial at −80°). The brain samples were obtained from The Netherlands Brain Bank, Netherlands Institute for Neuroscience, Amsterdam (open access: www.brainbank.nl). All Material has been collected from donors for or from whom a written informed consent for a brain autopsy and the use of the material and clinical information for research purposes had been obtained by the Netherlands Brain Bank.

Mice used in this study were bred in the Experimental Animal Facility of the Leiden University Medical Center and kept in individually ventilated cages with 12 hours of light/dark cycles at 20.5 °C and had ad libitum access to standard RM3 chow (SDS, Essex, UK) and water. All experiments were approved by and performed following the guidelines of the Animal Experiment Committee of the Leiden University Medical Center. Care was taken to limit the burden and distress for the animals as much as possible. The cortex and cerebellum of two 24-week-old female C57BL/10cSnJ wildtype mice were isolated and snap frozen on dry ice. For total RNA isolation, tissue was disrupted in tubes with MagNALyser Green Beads (Roche Diagnostics, The Netherlands) with TriPure Isolation Reagent (Roche Diagnostics). Isolation was performed with chloroform, and RNA was precipitated with isopropanol. The NucleoSpin RNA II kit including DNase digestion (Bioke, Leiden, The Netherlands) was used for RNA purification. RNA quality and concentrations were checked with the NanoDrop. RNA (1 µg) was used for cDNA synthesis with random hexamer primers. Expression of Dp427c, Dp427p, Dp140 and Dp71 (primer sequences available in Supplementary Table 8) was determined by SYBR Green-based real-time quantitative PCR (95 °C for 10 s, 60 °C for 30 s, and 72 °C for 20 s, 45 cycles followed by melting curve analysis) on the Roche LightCycler 480 (Roche Diagnostics). Samples were tested in triplo on a 384 wells plate and H_2_O and –RT controls were included for all primers. Housekeeping gene *RPL22* was used as a reference gene. Primer efficiencies were determined and analysis was performed with LinREgPCR^72^.

### Data availability

All data generated or analysed during this study are included in this published article (and its Supplementary Information files).

## Electronic supplementary material

Supplementary Information

Supplementary Tables 2-6
