# Author Correction: Timing and localization of human dystrophin isoform expression provide insights into the cognitive phenotype of Duchenne muscular dystrophy

**DOI:** 10.1038/s41598-018-22154-7

**Published:** 2018-03-01

**Authors:** Nathalie Doorenweerd, Ahmed Mahfouz, Maaike van Putten, Rajaram Kaliyaperumal, Peter A. C. t’ Hoen, Jos G. M. Hendriksen, Annemieke M. Aartsma-Rus, Jan J. G. M. Verschuuren, Erik H. Niks, Marcel J. T. Reinders, Hermien E. Kan, Boudewijn P. F. Lelieveldt

**Affiliations:** 1 C.J. Gorter Center for High Field MRI, Department of Radiology, Leiden University Medical Center, Leiden, The Netherlands; 2 Department of Neurology, Leiden University Medical Center, Leiden, The Netherlands; 3 Division of Image Processing, Department of Radiology, Leiden University Medical Center, Leiden, The Netherlands; 4 Delft Bioinformatics Lab, Delft University of Technology, Delft, The Netherlands; 5 Department of Human Genetics, Leiden University Medical Center, Leiden, The Netherlands; 6 Leiden Institute for Brain and Cognition, Leiden University, Leiden, The Netherlands; 7 Department of Neurological Learning Disabilities, Kempenhaeghe Epilepsy Center, Heeze, The Netherlands; 8 Department of Neurology, Maastricht University Medical Center, Maastricht, The Netherlands; 9 John Walton Muscular Dystrophy Research Centre, Newcastle University, Newcastle Upon Tyne, United Kingdom

Correction to: *Scientific Reports* 10.1038/s41598-017-12981-5, published online 03 October 2017

In this Article, some of the wording in the Results section is ambiguous and should be corrected as follows:

“In contrast to previous reports^18,29^, the Purkinje isoform Dp427p was virtually absent in the brain throughout development, with expression levels even lower than muscle dystrophin Dp427m.”

should read:

“In contrast to previous reports on mouse brain^18, 29^, the Purkinje isoform Dp427p was virtually absent in human brain throughout development, with expression levels even lower than muscle dystrophin Dp427m.”

In addition

“Yet in line with previous studies^30,31^, Dp427p was expressed in the mouse cerebellum and not in the mouse cerebral cortex.”

should read:

“Yet in line with previous studies^29,30,31^, Dp427p was expressed in the mouse cerebellum and not in the mouse cerebral cortex.”

