# Supplementary material for: Timing and localization of human dystrophin isoform expression provide insights into the cognitive phenotype of Duchenne muscular dystrophy
Source: Sci Rep. 2017 Oct 3;7:12575. doi: 10.1038/s41598-017-12981-5 (PMC5626779; doi:10.1038/s41598-017-12981-5)
Supplement: Supplementary file 1 — Supplementary Information [file 41598_2017_12981_MOESM1_ESM.pdf]

Supplementary Information

Timing and localization of human dystrophin isoform expression provide insights into the cognitive phenotype of Duchenne muscular dystrophy

Nathalie Doorenweerd[*], Ahmed Mahfouz, Maaike van Putten, Rajaram Kaliyaperumal, Peter A.C. ’t Hoen, Jos G.M. Hendriksen, Annemieke M. Aartsma-Rus, Jan J.G.M. Verschuuren, Erik H. Niks, Marcel J.T. Reinders, Hermien E. Kan, Boudewijn P.F. Lelieveldt

*Corresponding author email: N.Doorenweerd@lumc.nl

This PDF file includes:

Supplementary Figure 1

Supplementary Table 1

Supplementary Table 7

Supplementary Table 8

Other Supplementary Material for this manuscript

Tables S2 – S6 (as Excel files)

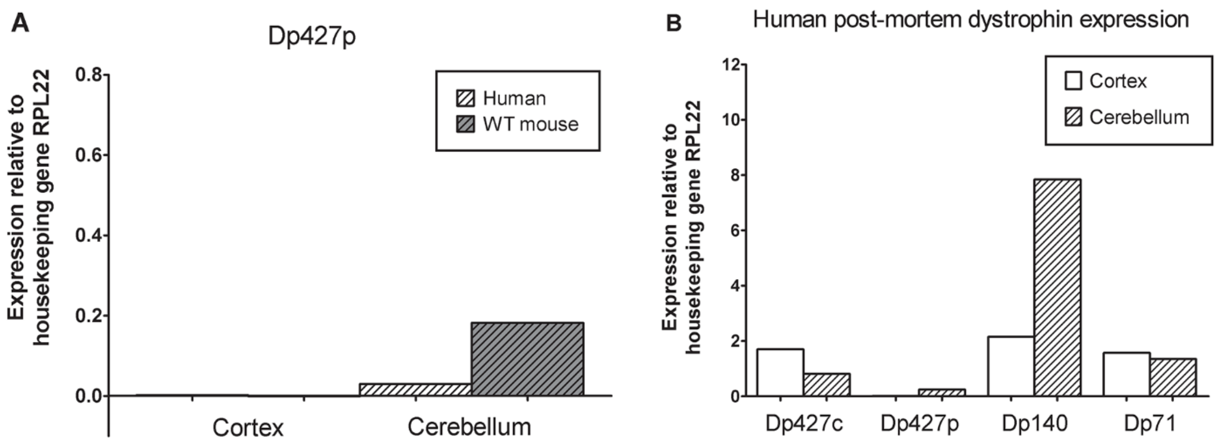

Supplementary Figure 1. Ex-vivo qPCR isoform expression. Dystrophin isoform expression qPCR results are depicted as a ratio with respect to housekeeping gene RPL22 to allow for comparison. Figure A shows expression of isoform Dp427p in the human and wild type (WT) mouse cortex and cerebellum. Figure B shows the expression of the isoforms Dp427c, Dp427p, Dp140 and Dp71 in the human cortex and cerebellum.

Supplementary Table 1. Overview of the donor brains included, per age and sex, divided into developmental stages

Developmental stage			Donors
1	8-9 pcw	Early fetal	8 pcw 1♂, 9 pcw 1♂
2	12-19 pcw	Early-mid fetal	12 pcw 3♀, 13 pcw 2♂ & 1♀, 16 pcw 3♂, 17 pcw 1♀, 19 pcw 1♀
3	21-37 pcw	Mid-late fetal	21 pcw 1♂ & 1♀, 24 pcw 1♂, 25 pcw 1♀, 26 pcw 1♀, 35 pcw 1♀, 37 pcw 1♂
4	4-12 mon	Infancy	4 mon 3♂, 10 mon 1♂, 12 mon 1♀
5	2-4 yrs	Early childhood	2 yrs 1♀, 3 yrs 1♂ & 1♀, 4 yrs 1♂
6	8-11 yrs	Late childhood	8 yrs 2♂, 11 yrs 1♀
7	13-15 yrs	Adolescence	13 yrs 1♀, 15 yrs 1♂
8	18-19 yrs	Early adulthood	18 yrs 1♂, 19 yrs 1♀
9	21-30 yrs	Young adulthood	21 yrs 1♀, 23 yrs 1♂, 30 yrs 1♂
10	36-40 yrs	Middle	36 yrs 1♂, 37 yrs 1♂, 40 yrs 1♀

Supplementary Table 7. Roadmap Epigenomics samples.

Sample number	Sample name	Sample annotation
1	Fetal Brain	Fetal Brain
2	Fetal Brain	
3	Neurospheres Cortex Derived	Adult Brain
4	Brain Hippocampus Middle	
5	Brain Substantia Nigra	
6	Brain Anterior Caudate	
7	Brain Cingulate Gyrus	
8	Brain Inferior Temporal Lobe	
9	Brain Angular Gyrus	
10	Brain Germinal Matrix	
11	Brain Mid Frontal Lobe	
12	NH-A Astrocyte Primary Cells	
13	Fetal_Muscle_Trunk	Fetal Muscle
14	Fetal_Muscle_Leg	
15	Fetal Heart	Fetal Heart
16	Skeletal Muscle	Adult Muscle
17	Skeletal Muscle	
18	Left_Ventricle	Adult Heart
19	Right_Ventricle	
20	Right_Atrium	
21	Aorta	
22	Adult Liver	Adult Liver
23	Fetal Kidney	Fetal Kidney

Supplementary Table 8. qPCR primer sequences

Target	Species	Forward primer (5'..3')	Reverse primer (5'..3')
RPL22	Human	tcgctcacctccctttctaa	tcacggtgatcttgctcttg
Dp427p	Human	cagcaaaaagctttcctatgaagg	aaattgtgcatttacccattttgtg
Dp427c	Human	aggagaaagatgctgttttgca	aaattgtgcatttacccattttgtg
Dp140	Human	tgctggctgctctgaactaa	gttctgacaacagtttgccg
Dp71	Human	ctgggaagctcactcctcca	tcccagcaagttgtttgagtc
Rpl22	Mouse	aggagtcgtgaccatcgaac	tttggagaaaggcacctctg
Dp427p	Mouse	tcacagcaactggagtggaa	aattgtgcatttatccattttgtga
